# Soil Fertility, Plant Nutrition and Nutrient Management

**DOI:** 10.3390/plants14010034

**Published:** 2024-12-26

**Authors:** Lin Tang

**Affiliations:** Department of Agroecology, Aarhus University, 8830 Tjele, Denmark; lin.tang@agro.au.dk

## 1. Introduction

Soil fertility refers to the ability of soil to sustain agricultural plant growth; fertile soil provides a habitat with essential nutrients and favorable chemical, physical and biological characteristics to sustain plant growth [1], without toxic substances that inhibit plant development [2]. Essential plant nutrients are critical for plant growth and reproduction, a deficiency of them causes the plant growth cycle to fail [3]. If the soil is cropped, it is necessary to supplement soil fertility by adding fertilizers to satisfy crop growth demand and increase or sustain yield [4]. Appropriate nutrient management is crucial to beneficial agronomic systems and environmentally safe crop production [5]. Considering the form of each element that plants absorb from soil, the relative amount of nutrients needed for optimum economic yield, the quantity removed by crop harvest, the nutrient transformations and interactions within the soil and the potential mobility of nutrients is advantageous for rational fertilization strategies [6,7]. Nutrient diagnosis via soil testing, plant analysis and crop canopy sensing, as the main methods, are tools for plant nutrition determination and crucial steps for the recommendation of fertilizers to crop producers or advisors [8,9].

In such a context, this Special Issue aims to enhance our understanding of the effects of environment and management on changes in soil fertility characteristics and the subsequent actions on nutrient absorption and utilization by plants, as well as to deliver approaches and strategies for nutrient management for improvements in fertilizer use efficiency and plant production. This Special Issue presents ten original scientific articles. The authors contributing to this Special Issue have affiliations with institutions from various countries including Brazil, China, Estonia, Finland, Greece, Latvia, Lithuania, Morocco, Spain and the USA. This resulted in an integral demonstration of diverse plant species (including vegetables, fruits, cereals, forages, trees and cover crops) grown in various culture systems (such as hydroponics, pot experiments and field trials) under different climates being studied at the physiological, molecular and ecological levels.

## 2. Advances in Soil Fertility, Plant Nutrition and Nutrient Management

The following is an overview of each paper, providing insights into how they collectively advance the development of soil fertility, plant nutrition and nutrient management research.

Xaxiri et al. [10] aimed to identify optimal nutrient management strategies for fruit vegetable species in closed-loop soilless culture systems. They hydroponically grew tomato, eggplant and cucumber in a floating system under identical cropping conditions and determined the uptake concentrations using mass balance models based on nutrient removal from nutrient solution and nutrient recovery in the plant tissues, respectively. The study accurately estimated the mean nutrient-to-water uptake ratio of each fruit vegetable species at its vegetative and reproductive stages. The results can be used to precisely adjust the nutrient supply in closed-loop soilless cultivation to the plant’s uptake, thus avoiding both the depletion and accumulation of nutrients in the root environment.

Liu et al. [11] performed a meta-analysis to evaluate the overall effects of organic amendments replacing chemical fertilizers on changing microbial activity and community structure in different agroecosystems on a global scale. The study found that organic amendments increased total microbial biomass, bacterial biomass, fungal biomass, Gram-positive bacterial biomass and Gram-negative bacterial biomass. Land use type, mean annual precipitation and initial soil pH were the essential factors affecting microbial activity response. In addition, there was a positive correlation between microbial biomass and microbial functionality. This research highlighted that organic amendment changed soil carbon and nutrient availability thus shifting microbial biomass and improving soil microbial functionality.

Li et al. [12] aimed to explore the relationship between organic waste and mineral nutrient utilization and maize production with straw return under nitrogen (SRN) treatment and reveal the underlying molecular mechanisms. The study found that SRN treatment increased maize grain yield, net photosynthetic rate, transpiration rate, its content of chlorophyll, soluble sugar and protein, as well as the activities of antioxidant enzymes, but reduced intercellular carbon dioxide (CO_2_) concentration and malondialdehyde content. In addition, SRN downregulated the transcript levels of promising genes involved in the transport and assimilation of potassium, phosphate (P) and nitrogen (N), as well as the metabolism of sugar, lipid and protein, suggesting that SRN improved maize production by regulating gene expression to reduce nutrient shortage stress and to enhance the photosynthesis of ear leaves at the grain-filling stage.

Nackley et al. [13] explored the novel application of tectonite dust in agriculture, revealing its potential as a sustainable soil amendment with economic and environmental benefits, via a three-step process. The study found that tectonite increased water-holding capacity and improved soil structure when added to bark substrates, increased wheat height and biomass in the small-scale short-term trial and increased wheat grain yield in the farm-scale full-season trial. Using tectonite as a soil amendment aligns with sustainability goals, reduces waste and greenhouse gas emissions, offers cost savings compared to synthetic fertilizers and stimulates local economies. This research highlighted the importance of utilizing waste byproducts in agriculture to achieve environmental and economic sustainability.

Vigricas et al. [14] monitored total soil CO_2_ efflux, environmental factors, soil physical and chemical parameters and soil water table level in drained nutrient-rich organic forest and perennial grassland soils in the growing seasons of two years. The study found that forest soil with a lower peat decomposition rate had higher soil organic carbon (C) and total N concentrations as well as C/N ratio; total CO_2_ efflux was 30% higher under perennial grassland than it was under forest stands; air and topsoil temperatures were the most important among all the environmental parameters in explaining the variation in total CO_2_ efflux from nutrient-rich organic soils. These results indicate that, from a climate change mitigation perspective, forest stands are a better land use option for reducing CO_2_ emissions than perennial grasslands.

Luo et al. [15] investigated the effects of straw returning and new fertilizer (slow-release and water-soluble fertilizers) substitution on rice production and soil properties in paddy fields. The study found that straw returning and new fertilizers increased rice yield, plant height, main root length, tiller number, leaf area index, chlorophyll content and aboveground dry weight, as well as N and P uptake, harvest indexes and partial productivity. Moreover, straw returning and new fertilizers improved soil physicochemical properties and enzyme activities. These results indicate that slow-release chemical fertilizers were the best fertilization method to improve rice production and enhance soil properties, providing useful information for better fertilization management for rice production in paddy fields.

Hnizil et al. [16] aimed to identify and refine agricultural practices that enhance crop performance at various growth stages while supporting sustainable farming objectives. They explored the interplay between N doses and seeding rates on wheat yield, biomass and protein content for five growing seasons, utilizing precision agriculture tools such as the Normalized Difference Vegetation Index, Soil–Plant Analysis Development and Canopy Temperature. The study found that an intermediate N dose combined with a moderate seed rate enhanced wheat yield. Additionally, reduced N levels increased protein content in wheat grain while high N application promoted growth during the tiller phase, early N application boosted chlorophyll content and optimal N and seed rates modulated plant stress responses. This research offered actionable recommendations that will help improve wheat farming efficiency and environmental sustainability and contribute to better food security and agricultural practices.

Liu et al. [17] conducted a meta-analysis to identify the long-term effects and the influencing factors of barren land afforestation on plant productivity, soil fertility and soil moisture on a national scale in China. The study found that the long-term benefits of afforestation in terms of enhancing plant productivity and soil fertility were limited. Additionally, climate factors (precipitation and humidity index) were crucial to enhancing plant productivity, while geographic factors (lower elevations and gentler slopes) were associated with improved soil fertility. This research highlighted the need for ongoing soil management and ecological maintenance in afforestation projects to sustain the soil fertility benefits, provides a robust scientific foundation for afforestation strategies aimed at barren land restoration and offers valuable insights for policy formulation in barren land afforestation.

Leite et al. [18] aimed to verify the effect of cover crops on soil P availability and use by successive plants and the accumulation of soil P in a no-tillage system. The study found that the accumulation of P in the shoot dry matter was highest in sunn hemp, while millet reduced labile inorganic P in the soil surface and increased moderately labile and non-labile organic P along the soil profile. In addition, systems using cover crops recovered all of the P fertilized in soybeans. This research indicates that the growth of cover crops modified the availability of P in the soil after a succession of crops under a no-tillage system, highlighting the biological incorporation of P at depth by the intense cultivation of plants.

Tassinari et al. [19] aimed to evaluate the effects of management strategies to increase P content in the soil profile and enhance grape production and maintain grape must composition by investigating P concentration in leaves at flowering and veraison, P content in soil, grape production and its components as well as the chemical parameters of grape must. The study found that P application was associated with soil mobilization and increased grape production, but the P application mode did not affect P concentration in the leaves or the chemical parameters of the grape must (except for the total anthocyanins). This research highlighted that P application and incorporation into the soil profile was an efficient strategy for increasing grape production in full-production vineyards.

## 3. Conclusions

In summary, this Special Issue highlighted the rapidly evolving scientific frontiers of soil fertility, plant nutrition and nutrient management; illustrated current research findings; and provided important theoretical foundations and practical pathways for addressing the increasingly complex challenges in this research field. As the Guest Editor, I am grateful to all the authors for choosing to publish their research in this Special Issue, and to the reviewers for maintaining the standards of the publications in this Special Issue. I hope that the presented articles will serve as a rich source of knowledge, inspiration and fresh perspectives on soil fertility, plant nutrition and nutrient management for both authors and readers alike.
